# The FLEX study school-based physical activity programs – measurement and evaluation of implementation

**DOI:** 10.1186/s12889-018-6335-3

**Published:** 2019-01-16

**Authors:** Catherine M. Wright, Virginia R. Chomitz, Paula J. Duquesnay, Sarah A. Amin, Christina D. Economos, Jennifer M. Sacheck

**Affiliations:** 1 Gerald J. and Dorothy R. Friedman School of Nutrition Science and Policy, Tufts University, 150 Harrison Avenue, Boston, MA 02111 USA; 2 Department of Public Health and Community Medicine, Tufts University School of Medicine, 136 Harrison Avenue, Boston, MA 02111 USA; 3 Department of Nutrition and Food Sciences, University of Rhode Island, 125 Fogarty Hall, Kingston, RI 02881 USA; 4 ChildObesity180, Tufts University, Boston, MA 02111 USA; 5 Department of Exercise and Nutrition Sciences, Milken Institute School of Public Health, The George Washington University, 950 New Hampshire Avenue, Washington, DC 20052 USA

**Keywords:** Physical activity, Children, School-based intervention, Program evaluation

## Abstract

**Background:**

Increasing children’s physical activity (PA) at school is critical to obesity prevention and health promotion. Implementing novel, low-cost PA programs offers potential to contribute to children’s in-school PA, particularly in resource-constrained schools. This evaluation describes implementation fidelity, reach, and dose of two PA programs in the Fueling Learning through Exercise (FLEX) Study.

**Methods:**

Thirteen diverse, low-income Massachusetts elementary schools were recruited and randomized to the 100 Mile Club walking/running program (*n* = 7) or CHALK/Just Move classroom activity break PA program (*n* = 6). Intervention programs were delivered across two school years. Surveys with program champions/teachers and children, in-session measurement of children’s PA by accelerometry (Actigraph GT3X) in a subset of schools, and key informant interviews were used to collect information on implementation, including fidelity, dose, reach, and sustainability, and to calculate an implementation score.

**Results:**

Six CHALK/Just Move schools implemented the program in both years. Two schools randomized to 100 Mile Club did not implement at all, and only three schools implemented both years. Implementing schools had similar implementation scores (range = 0–3; 100 Mile Club = 2.0 vs. CHALK/Just Move = 1.9) but fidelity to core and enhanced elements differed between programs. In 100 Mile Club schools, dose of program delivered was greater than in CHALK/Just Move schools (34.9 vs. 19.7 min per week). Dose of PA received per session was also greater in 100 Mile Club schools (*n* = 55, 2 schools) compared with CHALK/Just Move schools (*n* = 160, 2 schools) (13.6 min vs. 2.7 min per session). A slightly higher proportion of eligible children participated in CHALK/Just Move compared to 100 Mile Club (54.0% vs. 31.2%). Both programs were well received by champions/teachers and students.

**Conclusions:**

Program implementation varied across programs and schools, and erosion in delivery was seen over the two years. However, among implementing schools, additional PA was delivered and received, and the programs were generally well-received. Although school resource issues remain barriers to implemention, this evaluation demonstrates that low-cost programs may enhance PA opportunities. Future research should evaluate how multiple programs can be implemented to increase children’s PA at school.

**Trial registration:**

ClinicalTrials.gov Identifier: NCT02810834. Registered May 11, 2015.

**Electronic supplementary material:**

The online version of this article (10.1186/s12889-018-6335-3) contains supplementary material, which is available to authorized users.

## Background

Physical activity (PA) plays a critical role in childhood obesity prevention in addition to conferring a number of other important health benefits [1–3]. However, fewer than half of all children in the U.S. meet the recommended 60 min of daily moderate-to-vigorous physical activity (MVPA) [4]. Schools are an ideal setting to provide opportunities to improve children’s PA, given the amount of time they spend in school. Although guidelines recommend that schools provide at least 30 min of daily MVPA [5], competing demands, including a crowded school curriculum, focus on standardized tests, and constrained budgets, have limited PA programming in schools [6, 7].

Experts have recently called for a “whole school” approach to increasing children’s PA [8], in which physical education (PE), recess, in-class PA breaks, and before- and after-school programs collectively promote a healthy school environment. Evidence suggests that this may be even more critical for socioeconomically disadvantaged children who often accrue a greater proportion of their total daily PA at school [9]. In addition, environmental barriers, such as limited PA-supporting policies, activities, and infrastructure, have been observed in lower-socioeconomic status (SES) schools [10, 11], which can further constrain school-time PA opportunities and exacerbate disparities in PA. Nevertheless, even small increases in school-time MVPA may lead to the accumulation of additional daily PA and contribute positively to academic outcomes [12].

Novel strategies are needed to increase PA opportunities for children at school, particularly among low-SES children. Teacher-developed, champion-led school-based PA programs may have unique advantages with respect to acceptability, feasibility, and sustainability, compared to programs developed by researchers outside the school environment [13]. Documenting real-world implementation of these school-based PA programs is critical to understanding how they can be successfully sustained and scaled despite resource and time constraints and competing priorities.

This paper describes the implementation of two innovative school-based PA programs in the Fueling Learning through Exercise (FLEX) study [14], a cluster-randomized-controlled trial designed to evaluate the impact of two programs, 100 Mile Club® and CHALK/Just Move™, on PA, cognition, and academic outcomes among elementary school children from low-income communities. The programs, identified through a nationwide contest, were developed by educators for use in schools and were selected for the trial based on their potential for scalability [15]. A primary aim of the FLEX Study was to evaluate the relative impact of these two programs on children’s school-time and total daily MVPA. The evaluation presented here sought to quantify the extent to which the programs, which by design are different, were implemented in study schools, and help explain primary outcome results.

Using a mixed methods approach, we aimed to understand key elements of implementation, including program reach, dose, and fidelity by engaging direct implementers and participants. In addition, we sought to identify barriers to and facilitators of program delivery to inform opportunities for future dissemination.

## Methods

### FLEX study design and program delivery

Detailed methods for FLEX are described elsewhere [14]. Eighteen schools from eight lower-income communities in Massachusetts were recruited, either by assessing initial interest at the district level or directly with school administration, and block-randomized to the 100 Mile Club (*n* = 7), or CHALK/Just Move (*n* = 6), or control (*n* = 5). Randomization occurred after schools agreed to participate and to implement the program to which they were assigned. The interventions were delivered across two school years (2015–2016 and 2016–2017) for children in grades 3 through 5. One 100 Mile Club school opened the program to all grades. Programs were available to all children in participating grades/classrooms regardless of whether they were enrolled in the trial. Table 1 describes program characteristics and implementation according to randomization. The Institutional Review Board at Tufts University approved the study.

**Table 1:** Description of PA program characteristics and implementation according to randomization

	100 Mile Club	Implementation	CHALK/Just Move	Implementation^a^
Underlying philosophy/origin	School-based walking/running program promoting goal setting to encourage students to walk/run 100 miles over the school year. *Core elements: offer ≥ 30 min per week, track miles* *Enhanced elements: display mileage progress, offer incentives, hold special event (family fun-run, school-wide event,* etc.*)*	Champions at schools delivered any program elements school-wide: Year 1: 5/7 schools Year 2: 3/7 schools	Classroom-based physical activity break program incorporating high and low intensity moves to be used throughout the school day. *Core elements: offer daily break of ≥ 5 min, use activity cards most or all of the time Enhanced elements: integrate breaks with academics, use student leaders*	Classroom teachers at schools delivered any program elements: Year 1: 6/6 schools (grades 3&4) Year 2: 6/6 schools (grades 3&4)
All Training (content, frequency, etc.)	Annual 30–40 min training offered to explain the program, assist with mapping routes, deliver program/tracking materials, bimonthly check-ins via email	Study staff provided training at schools: Year 1: 7/7 schools Year 2: 3/7 schools	Annual 10–20 min training offered to explain the program and deliver program guide and cards, bimonthly check-ins via email with newsletters to encourage engagement and implementation	Study staff provided training at schools: Year 1: 36/38 teachers trained Year 2: 12/42 teachers trained (some trained both years)
Materials/supplies provided	100 Mile Club program guide, clipboard box to assist with tracking in field, tracking posters, 1000 popsicle sticks for tracking laps	Study staff provided training at schools: Year 1: 7/7 schools Year 2: by request	CHALK/Just Move program guide and set of 24 activity cards	Study staff provided training at schools: Year 1: 38/38 Grade 3&4 teachers Year 2: 19/19 Grade 5 teachers; 4th grade teachers by request

^a^CHALK/Just Move data were from all six randomized and implementing schools

#### 100 Mile Club and CHALK/just move programs

100 Mile Club [16] is a program that encourages children to walk, jog, or run 100 miles over the course of the school year (approximately 3 miles per week). The program can be implemented before, during, and/or after school, and is led by one or two school-wide champions (e.g., PE teacher, administrator, etc.) who log children’s miles. Two core elements were central to the program: schools were asked to offer the program at least 30 min per week, which is sufficient for children to accrue 100 miles over the course of the school year, and to track mileage during sessions. Enhancements to promote participation included encouraging champions to display individual and school progress in a prominent location in the school, providing incentives at milestones (e.g. tee shirt at 25 miles), and holding special events (e.g. fun run, school-wide parade).

CHALK/Just Move [17] is a program of structured classroom-based PA breaks that combines high- and low-intensity movements (e.g. jumping jacks, squats, yoga poses) to provide PA for children while learning. CHALK/Just Move activities are presented on cards with pictures demonstrating movements and suggestions for connecting moves to academic subjects like math, English language arts, and science. Two core elements were essential to the program: classroom teachers were asked to use the cards during most or all sessions and offer at least one daily session of about 5 min, which was determined to be feasible given teachers’ busy schedules. Program enhancements designed to promote children’s and teachers’ engagement included integrating breaks with academic material (e.g. practicing multiplication through jumping jacks) and having students lead breaks.

#### Champion and teacher training

FLEX study staff trained champions, who were typically tapped by the principal, and classroom teachers to implement the programs in both study years. In 100 Mile Club schools, study staff offered a 30–40 min training at school at baseline (year 1) to explain the program, helped champions identify the best outdoor and indoor routes, and delivered printed program guides and other materials. At the start of year 2, study staff conducted check-ins via phone to re-engage champions, helped troubleshoot any barriers that surfaced during the first year, and offered support and/materials as needed. In CHALK/Just Move schools, study staff offered 10–20 min trainings annually to classroom teachers who would be implementing the program and delivered program guides and activity cards. Study staff provided support to champions and teachers throughout the two-year intervention, including regular check-ins via email. All schools received a $1000 stipend to support their respective program.

### Intervention implementation evaluation methods

The primary aims of the evaluation, informed by a process evaluation framework [18], were to document and quantify the quality and quantity of programming that schools delivered (implementation fidelity), to track the number of students who participated in the programs (reach), and to tally the average number of program minutes offered per week (dose delivered) and PA accrued in a given session (dose received). Program activities and activity benchmarks were specific to each individual program and by design were not intended to be equivalent. In addition, we sought to document receptivity to the programs, from the perspectives of both teachers/champions and students, as a measure of sustainability and scalability. Evaluation of the two programs could not be completely parallel due to differences in the structure and implementation of 100 Mile Club, a multi-grade program led by a single champion, and CHALK/Just Move, a classroom-based program. Analytic metrics are described for each evaluation measure in Table 2.

**Table 2:** Description of measures used to assess PA program implementation

Process measures	Implementation factor(s) measured	100 Mile Club	Collected/conducted	CHALK/Just Move	Collected/Conducted^a^
Program surveys *Teacher and champion (web-based survey link sent to all teachers & champions at each time point)*	Implementation and fidelity Dose delivered Reach Receptivity and sustainability	Midpoint (Year 1), Post-Intervention (Year 2)	Baseline: 6/7 schools Midpoint: 5/7 schools Post-intervention: 3/7 schools	Midpoint (Year 1), Post-Intervention (Year 2)	Baseline: 16/57 teachers/classrooms Midpoint: 6/23^b^ teachers/classrooms Post-Intervention: 20/57 teachers/classrooms
Child surveys *Conducted at post-intervention to assess participation and reception of program*	Receptivity	Post-Intervention (Year 2)	Post-intervention: 5/7 schools	Post-Intervention (Year 2)	Post-intervention: 6/6 schools
Accelerometry *In-session physical activity measurement*	Dose received	Physical activity measured by accelerometer; start and end time of session recorded by research staff	2 implementing schools	Physical activity measured by accelerometer; start and end time of session(s) recorded by classroom teachers	2 implementing schools, 8 classrooms
Key informant interviews *Conducted with champions and teachers after post-intervention*	Implementation and fidelity Receptivity and sustainability	20–30 min interviews conducted via telephone by research staff	4 champions	20–30 min interviews conducted via telephone by research staff	14 classroom teachers

^a^CHALK/Just Move data are from all six randomized and implementing schools

^b^Only 4th grade teachers may have participated for two years and therefore were only ones asked to complete a survey at midpoint

#### Program surveys

Champions and teachers were asked to complete brief online surveys at midpoint, to report details of year 1 implementation and post-intervention, to report details of year 2 implementation and plans for continuing programming). For 100 Mile Club schools, surveys included questions about scheduling of program sessions, numbers of participants, and methods for tracking and displaying mileage. For CHALK/Just Move schools, surveys included questions about the number and duration of daily breaks, use of activity cards, and integration of PA breaks with academic material. Champions and teachers were sent links to online surveys via email. Study staff sent reminders to non-responders.

#### Child surveys

Consented child participants randomized to 100 Mile Club or CHALK/Just Move were asked to complete a brief post-intervention survey to assess their participation in and feelings about the program. Questions asked children to report whether or not they participated in the program, whether they liked it, and whether they would participate again.

#### In-session assessments

In spring 2017, assessments were conducted in a subset of implementing schools to measure the time children spent engaging in PA during program sessions (dose received). All program schools were invited to participate. Prior to conducting assessments, research staff sent information letters home to parents explaining the evaluation and providing them the opportunity to “opt out” for their child. Likewise, children were given the opportunity to decline to wear an accelerometer prior to the start of assessments. Children who agreed were outfitted with waist-worn accelerometers (GT3x + and wGT3X-BT models, Actigraph LLC, Pensacola, FL) by trained study staff according to a standard protocol [14] [19]. In 100 Mile Club schools, children were outfitted with accelerometers at the beginning of sessions; study staff noted session start and end times. In CHALK/Just Move schools, children were outfitted with accelerometers at the beginning of the school day and teachers were asked to record start and end times of any breaks held that day on a form provided by and returned to research staff. Devices were collected at the end of sessions (100 Mile Club) and the end of the school day (CHALK/Just Move).

#### Key informant interviews

In Fall 2017, after completion of the study, semi-structured interviews with champions and teachers at schools randomized to either 100 Mile Club or CHALK/Just Move were conducted by telephone by a trained research assistant (see Additional files 1 and 2: Appendix for interview guides). Interviews were requested of all 100 Mile Club champions. In CHALK/Just Move schools, we initially contacted all fourth grade teachers as they were asked to implement the program both years. The 20–30 min interviews were designed to expand on the quantitative surveys and contextualize those results by delving more deeply into specifics of how programs were implemented including scheduling and timing. We sought to document how programs were received, and factors related to program sustainability and scalability, including perceptions of which elements were most and least successful, challenges to implementation, and ideas on how programs could be improved to better engage teachers and children. Interviews were recorded and transcribed, and assessed for emergent themes. Those interviewed were provided a $35 gift card for participation.

### Intervention evaluation scoring development and analysis

Intervention implementation was evaluated at two levels. The first level of the evaluation was aimed at identifying which schools randomized to 100 Mile Club or CHALK/Just Move implemented the program at all (randomized cohort). This was based on study staff’s direct contact with school staff and inquiries regarding PA program implementation. For the next evaluation level, implementation metrics were applied to schools that implemented any program elements during the first year of FLEX (implementation cohort); metrics assessed the two-year average fidelity and dose of the program and the maximum reach for either year for each program Descriptive analyses were used to summarize implementation results for both programs.

For the implementation cohort, data from champion and teacher surveys were extracted from Qualtrics and analyzed using Microsoft Excel. Implementation details and dose were reported for 100 Mile Club schools by champions. CHALK/Just Move implementation details and dose were reported by teachers and averaged across classrooms to create school-level metrics to parallel 100 Mile Club. Interview and observation data were used to supplement incomplete survey data.

#### Implementation and fidelity

We estimated the percentage of schools that offered core elements of the PA program over two years as well as the percentage of schools that offered any enhancements. An implementation score was created that ranged from 0 to 3. This score is comprised of the sum of two separate scores: fidelity to core elements (0–2) plus the use of enhanced program elements (0–1). Schools could receive a score of 0 for not implementing at all, a score of 1 for not implementing core elements to standard, or a score of 2 for fully implementing both core required elements (reported as “most of the time” or “always” on surveys). A score of 0 was assigned if no enhancement was used at least “most of the time” and a score of 1 was given when at least 1 enhancement was used “most of the time” or “always.” A key rationale for developing an overall implementation score was to have a metric to compare these two programs, however different, across the entire study to quantify delivery and explain primary outcomes discussed in separate manuscripts.

#### Dose delivered

Dose of programming delivered was defined as minutes per week per school. Champions and teachers were asked to report the minutes per week that they offered PA programming at midpoint (year 1) and post-intervention (year 2) as categorical responses. For 100 Mile Club, champions selected the response that reflected total minutes of PA programming offered each week across all sessions. Categories, in minutes, were 0–15, 16–30, 31–45, 46–60, 61–75, 76+. CHALK/Just Move teachers were asked to report sessions offered per week and minutes per session. To estimate total PA minutes per week, the medians of the selected categorical response for each question were multiplied to determine an estimated PA program minutes per week. Responses for sessions per week were 0–2, 3–4, 5–6, 7–8, 9–11, 12+. Minutes per session responses were 0–2, 3–4, 5–6, 7–8, 9–10, 11+. For example, if a teacher reported 3–5 (median = 4) sessions per week and 7–8 (median = 7.5) minutes per session, then 30 min of weekly PA programming was estimated. If 12+ sessions or 11+ minutes were reported, 12 sessions and 11 min were used as there was no median value.

#### Dose received

Dose received was determined by average minutes children engaged in light PA (LPA) and MVPA in program sessions, measured by accelerometer, in the subset of participating schools. Accelerometer data were processed using a 15-s epoch to more accurately record short, intermittent bursts of activity common among children [20]. Data were categorized into minutes of sedentary, light, moderate, and vigorous activity using thresholds developed specifically for children [21], and analyzed using Stata/SE 14.0 (College Station, TX).

#### Reach

To measure program reach, information reported in champion and teacher surveys was used. 100 Mile Club champions reported the number of students regularly participating across all grades including those either eligible for or enrolled in FLEX during year 1 (grades 3 and 4) and year 2 (grades 4 and 5). For one 100 Mile Club school where participant numbers were not provided on the survey, session participation observed and documented by research staff was used. CHALK/Just Move teachers reported whether their class was participating and the number of students in their class.

Reach was calculated by dividing the number of participating students by the total number of students in the eligible grades. The number of students in each grade at each school was obatined from publicly available data from the Massachusetts Department of Education (DOE) [22]. Reach was calculated at each school for years 1 and 2 and the highest percentage from either year was used. This method was chosen because there was not a clear way to know if the same or different students were participating each year.

#### Receptivity and sustainability

To better understand program receptivity and potential for sustainability and scalability for future dissemination, as well as barriers to and facilitators of implementation, we evaluated champion/teacher and student attitudes toward the programs. For champions/teachers, we defined receptivity as the percent who indicated on surveys at the mid-point and post-intervention time point that they would implement their respective program again. Children’s receptivity to the program was determined by both the percent of children who indicated on surveys that they liked the program and the percent of children who indicated that they would participate in the program again. Additional information on program sustainability was gathered from key informant interviews with champions and teachers from all schools randomized to the programs. Common as well as program-specific themes from the interviews were identified and refined by study staff familiar with program implementation.

## Results

Demographic characteristics of the FLEX schools as randomized are presented in Table 3. These data were obtained from publicly available data from the DOE [22]. The schools randomized to 100 Mile Club and CHALK/Just Move are similar: more than half of the students are non-white (62.6% non-white at 100 Mile Club schools; 58.9% non-white at CHALK/Just Move schools) and more than a third are from economically disadvantaged families [23] (39.2% at 100 Mile Club; 45.0% at CHALK/Just Move schools). Demographic characteristics of schools that implemented 100 Mile Club were similar to the full randomized group, including two schools that did not implement the program.

**Table 3:** Baseline characteristics of FLEX Schools

	100 Mile Club - randomized	100 Mile Club - implemented	CHALK/Just Move^a^
	(*N* = 7)	(*N* = 5)	(*N* = 6)
Eligible students per school (Mean, range)^b^	182 (95–509)	200 (107–509)	138 (47–180)
Race/ethnicity			
% African-American	19.1%	19.9%	8.3%
% Asian	9.9%	10.8%	6.5%
% Hispanic	28.4%	22.3%	40.7%
% White	37.2%	41.3%	41.1%
% Multi-race	5.1%	5.6%	3.3%
% other	0.1%	0.1%	0.1%
% economically disadvantaged^c^	39.2%	38.0%	45.0%
Per child school expenditures^d^ (mean, range)	$15,258 ($13,098–$20,312)	$14,679 ($13,098–$16,871)	$14,814 ($13,244–$16,871)

^a^All six randomized CHALK/Just Move schools implemented to some capacity

^b^Eligibility for program participation was grade 3–5, with exception of one school that made program available to grades K-5

^c^Economically disadvantaged measure based on student’s participation in the Supplemental Nutrition Assistance Program (SNAP), Transitional Assistance for Families with Dependent Children (TAFDC), Department of Children and Families’ (DCF) foster care programs, and/or MassHealth (Medicaid)

^d^Data from 2016

**Fig. 1:**
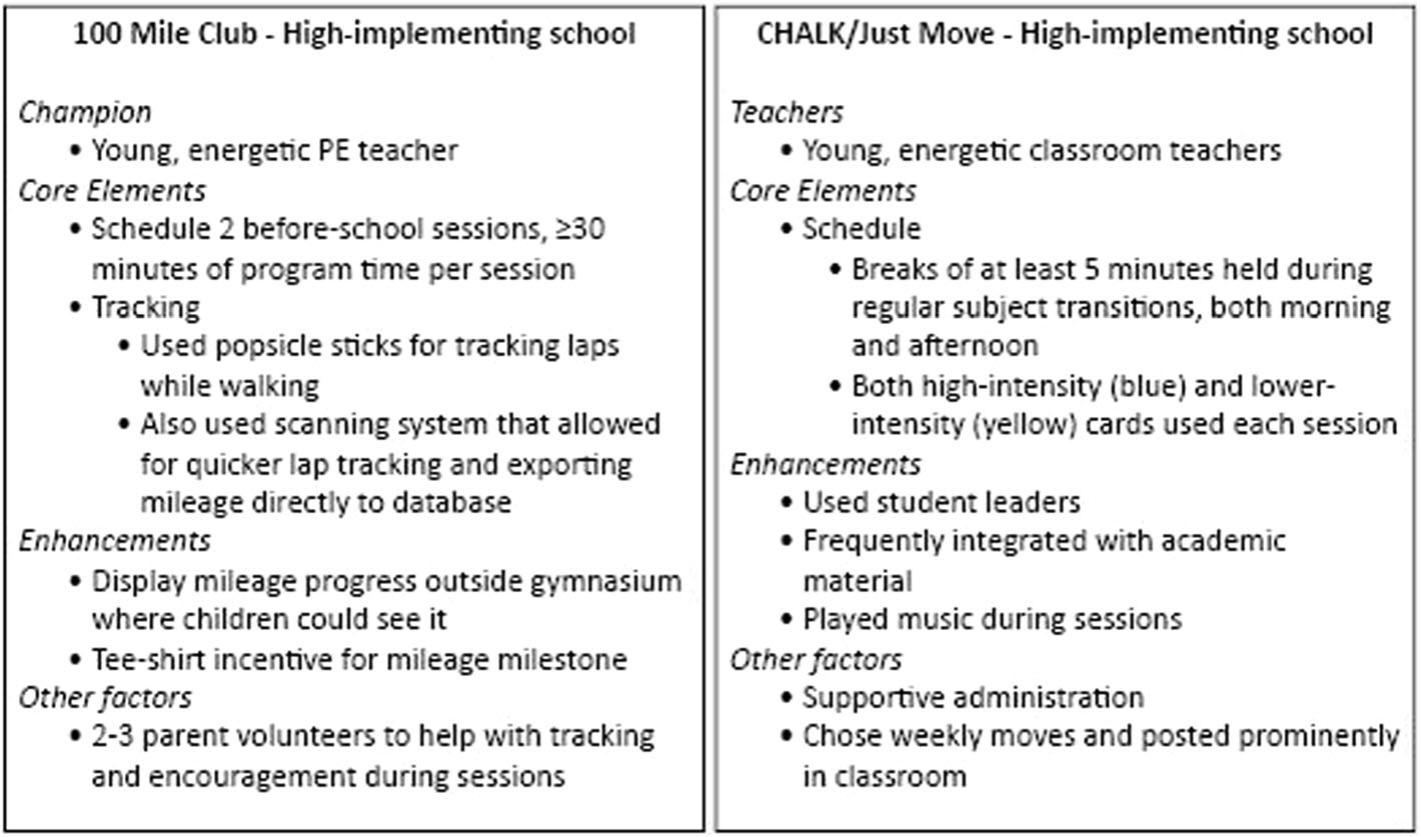
Champion/teacher characteristics and elements of exemplary program delivery for 100 Mile Club and CHALK/Just Move

Table 4 summarizes implementation metrics for all schools randomized to the FLEX programs (randomized cohort) and for schools that implemented the programs (implementation cohort). Figure 1 describes delivery in one school from each program with exemplary implementation, including core elements and enhancements.

**Table 4:** Summary of school-level measures estimated over two years among FLEX PA program schools

	100 Mile Club	CHALK/Just Move
Randomized cohort of FLEX schools:	*N* = 7	*N* = 6
Any implementation Year 1	71.4%	100%
Any implementation Year 2	42.9%	100%
Implementation cohort of FLEX schools	*N* = 5	*N* = 6
CHAMPIONS/TEACHERS	*n* = 5	*n* = 26
^a^Implementation		
% of schools offering core PA program elements to standard	40.0%	19.2%
% of schools offering any enhanced PA program elements	60.0%	65.4%
Implementation score per school (mean, range)	2.0 (1.5–3)	1.9 (1.5–2.33)
^b^Dose Delivered		
PA program minutes offered per week per school (mean, range)	34.9 (23.0–45.5)	19.7 (11.1–39.4)
^c^Dose Received	*n* = 55 students	*n* = 160 students
MVPA minutes (mean, SD) per session	9.6 (4.2)	0.5 (0.8)
LPA minutes (mean, SD) per session	4.0 (3.0)	2.3 (1.7)
Total PA minutes (mean, SD) per session	13.6 (5.0)	2.7 (2.2)
Sedentary minutes (mean, SD) per session	0.4 (1.3)	4.0 (2.7)
Average length of session in minutes (mean, range)	25.0 (20.0–30.0)	6.2 (3.0–11.0)
^d^Reach		
% of students reached per participating school (range)	31.2% (16.7–62.5%)	54.0% (34.4–100%)
Mean number of students reached per participating school	59	67
^e^Receptivity	*n* = 3	*n* = 19
% of champions/teachers who indicated they would do program again	66.7%	89.5%
STUDENTS	*n* = 285	*n* = 241
Receptivity		
% of students who indicated they liked the program	72.7%	69.2%
% of students who indicated they would do it again	80.8%	91.6%

^a^Implementation Score = Score (0–3) based on estimate of the extent to which essential and enhanced PA program elements were implemented to standard per school (see Table 1 for detail)

^b^PA Program minutes offered per week = based on estimated number of minutes that students were exposed to PA program per week per school averaged across two years of implementation

PA Program minutes planned per week = based on estimated number of minutes that champions/teachers reported planning to offer of PA programming (at baseline prior to program start)

^c^Average minutes children engaged in MVPA during program session; 100 Mile Club represents 2 schools, CHALK/Just Move represents 8 classrooms from 2 schools

^d^Reach = estimate based on maximum number of students exposed to the PA program per school per two years of implementation, estimate based on reported number of participants from surveys and for 1 school from direct observation

^e^Champion/teacher receptivity: 100 Mile Club represents 3 schools, CHALK/Just Move represents 19 teachers from 5 schools

### Randomized cohort

Of the seven schools randomized to 100 Mile Club, five (71.4%) schools in year 1 and three of these schools (42.9%) in year 2 implemented the program to some extent. Two schools (28.6%) did not implement the program either year. Six of six schools assigned to CHALK/Just Move implemented the program to some extent in each of the two years.

### Implementation cohort

The implementation cohort consists of the five 100 Mile Club schools and 26 classrooms from all six randomized CHALK/Just Move schools. Implementation metrics, reach, and dose, as well as teacher/champion and student receptivity to the programs, are presented in Table 4, and discussed in more detail below.

### Implementation and fidelity

Schools implementing the programs had overall similar implementation scores (100 Mile Club = 2.0 vs CHALK/Just Move = 1.9). However, fidelity to core and enhanced elements was different between the two programs. Champions at 100 Mile Club schools were somewhat more likely to report fidelity to core program elements (40.0% of respondents) than were teachers in CHALK/Just Move schools (19.2% of the respondents). However, about 65% of CHALK/Just Move teachers (17 of 26) offered at least one program enhancement, compared with 60% of the 100 Mile Club respondents. One 100 Mile Club school implemented fully to standard, with both core and enhanced elements. By comparison, no CHALK/Just Move school implemented fully to standard.

### Dose delivered

Champions in 100 Mile Club schools reported offering a higher dose of PA programming than did teachers in CHALK/Just Move schools. 100 Mile Club champions reported offering about 35 min per week. On average, 100 Mile Club schools held 3.4 sessions per week (data not shown). CHALK/Just Move teachers reported offering about 20 min per week per school. On average, across all CHALK/Just Move schools, teachers reported offering 3.6 sessions per week (data not shown).

### Dose received

100 Mile Club sessions were measured at two schools. In both cases sessions were held before school in a drop-in format where children joined as they arrived to school. Children (*n* = 55) from the two 100 Mile Club schools engaged in an average of 13.6 min (SD = 5.0) of PA per session. 100 Mile Club sessions were, on average, 25.0 min long (range: 20.0–30.0). In the two CHALK/Just Move schools, children (*n* = 160) from eight classrooms, engaged in an average of 2.7 min (SD = 2.2) of PA per session, with an average of 1.5 sessions (range: 1–3) per day. The average length of sessions was 6.2 min (range: 3.0–11.0). The impact of the programs on children’s overall PA will be presented in a separate manuscript.

### Reach

We estimated that the FLEX PA programs reached about one-third to half of all eligible students in participating schools at some point during the two years of implementation. A somewhat higher proportion and number of students participated in CHALK/Just Move (54.0%; *n* = 67 students per school) than 100 Mile Club (31.2%; *n* = 59 students per school).

### Receptivity, sustainability and scalability

In post-intervention surveys, the majority of champions and teachers reported that they would do their assigned program again. However, nearly 90% (17 out of 19) of CHALK/Just Move teachers, compared with 67% (2 out of 3) of 100 Mile Club champions reported that they would do the program again. Among children who consented to take part in the trial and who participated in the programs, 100 Mile Club was received somewhat more favorably with 72.7% responding that they liked the program compared with 69.2% of children who said they liked CHALK/Just Move. Children participating in CHALK/Just Move reported being more likely to do the program again compared with those who participated in 100 Mile Club (91.6% vs. 80.8% respectively).

Table 5 presents the results of post-intervention interviews with champions/teachers regarding program sustainability and scalability. Semi-structured interviews were conducted with champions from four 100 Mile Club schools, including one randomized, non-implementing school, and 14 CHALK/Just Move teachers, representing all six randomized and implementing schools. Champions at the three other 100 Mile Club schools did not respond to repeated interview requests. Interview participants generally expressed satisfaction with the programs. They highlighted the importance of PA for improving children’s focus at school and/or overall academic performance, and saw potential in these programs for conferring these benefits.

**Table 5:** Lessons learned about receptivity and sustainability

Common enabling factors across programs		
Adequate time available	Administration buy-in and support	Enthusiasm from implementer
Program specific enablers and barriers	100 Mile Club	CHALK/Just Move
Enablers	● Adequate outdoor/indoor routes	● Easy to use
	● Highly engaged school champion and staff	● Highly adaptable to class schedule
	● Delegation of tracking	● Flexibility with high and low intensity moves
		● Visual cues of cards helpful
		● Teachers sharing tips between themselves
		● Useful for classroom/subject transitions
Barriers	● Challenges making indoor route feasible and fun	● Physical cards can get lost
	● Conflicts with the school schedule-recess or PE	● Lack of language translation
	● Maintaining tracking	● Physical size of classroom for certain exercises
	● Responsibility for keeping program going falls mainly to one person	● Engagement of teachers and students
		● Lack or insufficient initial and ongoing training
Recommendations for future implementation	100 Mile Club	CHALK/Just Move
Sustainability		● More teacher training and/or check-ins
Scalability	● Define it as a school-wide program	● Translation to encourage ELL/ESL student leaders
	● Help schools enhance indoor routes	● Creation of cards with moves for older grades

Key themes specific to each program emerged from the interviews. Champions in 100 Mile Club schools indicated that before-school sessions were most successful and well attended, and a positive outcome of the program was that it promoted school community by facilitating interaction among children from multiple grades and encouraging participation of other school staff and/or parents, caregivers, and other family members. Teachers from CHALK/Just Move schools noted that the program was easy to integrate into the classroom and was often used as a transition between subjects. They also observed that integration of an online or video component could be beneficial, and having activity cards in additional languages would help engage students whose first language is not English.

## Discussion

This evaluation was conducted to describe the extent to which two school-based PA programs designed to increase student’s access to PA in school were implemented in FLEX, and to understand key factors associated with implementation that could inform future dissemination of the programs. The 100 Mile Club and CHALK/Just Move programs were identified through a nationwide crowdsourcing competition for their potential for scalability, including cost-effectiveness and flexibility [15] and determined to hold promise as low-cost, non-resource-intensive PA programs that may be suitable for lower-income, racially and ethnically diverse communities. The FLEX research design emphasized a real-world implementation, in which participating schools were not expected to allocate money to the programs, though they did require human capital and a suitable physical environment. Schools were assigned to PA programs based on the study randomization, rather than on their enthusiasm for implementing a specific program.

Results from this evaluation indicate that among our sample of diverse, low-to-moderate income schools that even when provided external resources and support to implement these programs, not all schools can or will do so. Among the seven schools randomized to 100 Mile Club, over a quarter of the schools did not implement the program at all, and among those that did there was erosion over the two years from 5 to 3 schools. Only one 100 Mile Club school champion implemented fully to standard and included enhancements in both years, like displaying children’s mileage progress outside the gym where they and others could see it, and recruiting parent volunteers (2–3 per session) to help with tracking and provide encouragement to the children during 100 Mile Club sessions (Fig. 1). Together, these types of factors likely worked to build children’s enthusiasm for and participation in the program.

In the case of CHALK/Just Move, all randomized schools implemented the program to some extent, but only 19% of classrooms did so to standard. In the example of the high-implementing CHALK/Just Move school (Fig. 1), teachers held daily breaks in both the morning and afternoon, regularly using them as transitions between subjects, and typically incorporated both high- and low-intensity moves. Sessions were enhanced by regularly choosing students to lead the breaks and by integrating them with academic material. While CHALK/Just Move may have been easier to implement overall, particularly as it did not rely on a single champion, not all teachers adopted to the same level and may not have sustained the same level of engagement throughout the school year.

While our ability to understand all implementation factors was limited by the relatively small number of respondents, it was clear that the programs were delivered inconsistently. The year-to-year erosion seen among the 100 Mile Club schools might suggest that though the program may have been relatively easy to implement initially, over time it proved challenging to sustain. Because the program typically relied on a single champion, the success of the program was driven by how engaged that champion was. Where it worked, it worked well, but this was not equally true for all schools. In addition, the school physical environment, in particular having adequate and accessible outdoor space, was key to successful and sustained implementation of 100 Mile Club. This may be especially relevant to urban and resource-strapped schools that have limited facilities available.

Despite the mixed results of implementation, among schools that implemented the programs, there was a reported additional dose of total school PA minutes delivered and received per week. This reported dose delivered was higher among 100 Mile Club schools (~ 35 min per week) than CHALK/Just Move schools (~ 20 min per week). Likewise, the measured average total PA dose received *per session* was higher in 100 Mile Club compared with CHALK/Just Move (13.6 vs. 2.8 min, respectively). This per session finding should be interpreted cautiously, since CHALK/Just Move sessions were delivered up to three times per day and up to five days per week, whereas 100 Mile Club sessions were usually delivered once per day and up to three times per week. Nevertheless, these findings suggest that both programs provided additional weekly PA minutes. Both programs were generally liked by the champions and teachers implementing the programs and by the children participating in them.

By design the FLEX Study was framed to evaluate two distinct PA programs chosen for their flexibility and adaptability to a range of school settings. The 100 Mile Club is a school-level, voluntary program typically held before school, while CHALK/Just Move is designed to be classroom-based. As a result, the two programs had differing reach in terms of number of children and, potentially, differences in participants by sex, weight status, and/or fitness. Unfortunately, we were not able to collect detailed information on all participating children. While the 100 Mile Club relied on a single champion to implement and maintain, responsibility for delivering CHALK/Just Move was spread across multiple teachers. Our evaluation found that because 100 Mile Club typically relied on one person to implement, champions were more likely to have either fully implemented or not implemented the program according to protocol; CHALK/Just Move had more teachers involved, but delivery of program elements was less uniform. As a result, the programs were offered in different ways across schools, and it was difficult to capture perfectly parallel implementation information for the two programs. Nevertheless, we collected enough similar information that we were able to compare and contrast the two programs. Inherent differences in the programs may therefore make one or the other more suitable to particular school environments or allow them to complement each other if implemented in tandem.

### Strengths and limitations

A key strength of this evaluation of the real-world implementation of the PA programs is that it was included as part of a large, randomized controlled trial. This allowed for collection of program implementation data from a range of school environments and, in the case of child survey data, from a large sample of participants.

Another strength of the FLEX evaluation is that the intervention programs were developed by schools and teachers and because participating schools were randomized to either 100 Mile Club or CHALK/Just Move, we were able to evaluate delivery of the programs in a quasi-real world setting. This is notable because though we assume that the schools that agreed to participate had at least some willingness to implement a new PA program, they were randomly assigned to the program, rather than choosing the one that might best fit their school environment or culture. The fact that schools were randomized to the programs allowed for opportunities to understand school- and champion/teacher-level factors that influenced delivery and may be critical for sustainability and scalability.

Additionally, a strength of this evaluation is the mixed methods approach. The use of both quantitative and qualitative methods allowed for a more detailed examination of the various aspects of implementation. Multiple dimensions of process evaluation were used at multiple time points during the study.

A limitation of this evaluation is that the sample of champion and teacher respondents to surveys and interviews was limited and did not capture demographic characteristics of the respondents themselves (i.e. age, years teaching, personal PA/fitness status). That information may be valuable in understanding how to develop champions in the future for these kinds of interventions [24]. Further, though the teachers and champions were the implementers and therefore in a strong position to report on what happened in the programs, there may have been response bias. This includes limited data from non-implementing schools and classrooms. Nevertheless, in these kinds of real-world studies, we must balance the needs of collecting all key study data with limiting the burden on schools and teachers on whom we rely to collect those data. Researchers must weigh the importance of collecting primary outcome data against the need to collect process data to better understand those outcomes. In addition, while direct observation of programming by researchers may afford collection of more objective data, the presence of researchers may also influence the dynamics of implementation.

In addition, though accelerometry provided an objective measure of in-session PA, the waist-worn device may not be sufficiently sensitive to record many of the CHALK/Just Move activities, particularly semi-stationary movements like stretches and yoga poses. Though schools that agreed to participate in the accelerometry sub-study were not necessarily representative of all schools, the sample was small, and data collected only one time, they did represent a range of implementation scores. Nevertheless, these data provide a picture of how much and what types of activity children engage in during the programs. In addition, by being integrated into the classroom setting, CHALK/Just Move may provide benefits beyond additional minutes of MVPA. Forthcoming analyses as part of FLEX will look at the impact of both programs on other aspects of children’s overall health and well-being, including cognitive function and academic achievement.

## Conclusions

The primary aim of the FLEX Study was to evaluate the impact of two different school-based PA programs on children’s PA and to explore their feasibility for dissemination and scalability, particularly for resource-constrained schools. This evaluation demonstrates that overall both programs were overall well received by schools, including both champions/teachers and students. Some schools were able to implement the programs with minimal resources and without extensive support from researchers, particularly in the first year. However, schools may need additional financial and human capital investment and/or policy support to promote long-term sustainability. Researchers must be cognizant of the fact that schools, with finite resources, are by necessity making tradeoffs in choosing to implement one program or another. The results of this evaluation suggest that both programs, if implemented, can enhance opportunities for PA at school. The 100 Mile Club shows potential for providing meaningful increased PA minutes for children, but may have limited reach as a voluntary, before-school program and the burden for implementation falls to one champion. CHALK/Just Move, as a classroom-based program, has the potential to reach a large number of children, with the burden for delivery spread across multiple teachers, but likely offers more limited benefits in terms of MVPA. Identifying the program or programs that best fit individual schools is necessary for securing the buy-in critical to their success. Future work should evaluate how multiple PA programs can be put in place in schools simultaneously to increase children’s opportunities for PA throughout the school day.

## Additional files

Additional file 1: FLEX Key Informant Interview Guide – 100 Mile Club. Key informant interview objective and guide for FLEX Study 100 Mile Club schools. (DOCX 18 kb)

Additional file 2: FLEX Key Informant Interview Guide – CHALK/Just Move. Key informant interview objective and guide for FLEX Study CHALK/Just Move schools. (DOCX 21 kb)

## Abbreviations

DOE: Department of Education
FLEX: Fueling Learning through Exercise
LPA: Light physical activity
MVPA: Moderate to vigorous physical activity
PA: Physical activity
PE: Physical education
SES: Socioeconomic status
