# Supplementary material for: The FLEX study school-based physical activity programs – measurement and evaluation of implementation
Source: BMC Public Health. 2019 Jan 16;19:73. doi: 10.1186/s12889-018-6335-3 (PMC6335723; doi:10.1186/s12889-018-6335-3)
Supplement: Supplementary file 1 — FLEX Key Informant Interview Guide – 100 Mile Club. Key informant interview objective and guide for FLEX Study 100 Mile Club schools. (DOCX 18 kb) [file 12889_2018_6335_MOESM1_ESM.docx]

**The FLEX Study**

**Key Informant Interviews**

**2015-2017 100 Mile Club Champions**

**Objectives and Guide**

**Objectives:**

- To assess overall satisfaction with the 100 Mile Club program
- To identify barriers to and enablers of implementation, including which program elements were most successful and to identify ways in which the program could be improved
- To identify other factors in school environment that may have impacted implementation of the program
- To gain insights into program sustainability and opportunities for scale

**Interview length:** 20-30 minutes

**Introduction (to be read by interviewer):**

Thank you for your involvement with the 100 Mile Club program at your school during the prior (two/one) school year(s). We would like to learn more from schools that implemented the 100 Mile Club, to understand which aspects of the program were successful and opportunities for improvement. We would also like to identify other factors in the school setting that may have impacted implementation of the 100 Mile Club. Finally we will ask you about your thoughts on how we might best bring this program to other classrooms.

The interview will take no more than 30 minutes. We will provide a $35 gift card as a thank you for your time. We are asking to record these interviews because we want to make sure we don’t miss any of your valuable feedback. Only Tufts University staff who are involved in this project will have access to the recordings. No identifying information, including your name, will be shared outside our working group, nor will it appear in any publication. You may choose not to answer any of the questions, and you may stop participating at any time. There are no direct benefits to you, but the information you provide will help us to scale and disseminate the 100 Mile Club program to other schools.

You may contact Dr. Jennifer Sacheck, the Principal Investigator leading this study at (617) 636-3917 for more information. If you have any questions about your rights as a research participant, you should contact Lara Sloboda, the IRB Operations Manager for Social, Behavioral & Educational Research at Tufts University at (617) 627-3276.

**VERBAL CONSENT (to be read by interviewer)**

Do you consent to proceed with the interview? Yes_____ No_____

*If yes:* do you agree to be recorded? Yes_____ No_____

*If yes:* we will start the interview now **(turn on recorder).**

*If no:* okay, we will start the interview now **(do not turn on recorder).**

**Discussion topics:**

1. Before I ask about your experience with 100 Mile Club can talk about what it was like to be asked to establish the program at your school?
2. We would like to get a sense of your overall satisfaction with the 100 Mile Club program. Can you say a few words about what it was like having the 100 Mile Club at your school during the previous two (one) school years?
3. Now I’d like to dig a bit deeper into some of the specific elements of the program and get a sense of whether they were a part of the 100 Mile Club at your school, as well as which of them you found to found to be more successful as well as those you may have found to be less successful.
   1. Can you tell me a bit more about the scheduling and timing of 100 Mile Club at your school?
      - Probe – What time of day and how many days of the week did you offer 100 Mile Club?
      - Probe – How long were the 100 Mile Club sessions?
      - Probe – When did the program get started and come to an end during the school year?
      - Probe (for non-adopters) – Was scheduling and/or participation a major barrier to getting the program off the ground?
   2. Can you tell me more about the system you used to track laps as well as total mileage throughout the program?
      - Probe – Did you track at the individual child level?
      - Probe – Did you display mileage progress anywhere or in some way acknowledge mileage progress?
   3. Can you tell me more about other elements you may have integrated into 100 Mile at your school?
      - Probe – Did you play music?
      - Probe – Did you have rewards for mileage completion?
      - Probe – Were there any announcements or other ways of recognition?
   4. Are there any other things you did as part of the 100 Mile Club at your school that you would like to tell us about?
      - Probe – Were any special 100 Mile Club events held?
      - Probe – Did you open up 100 Mile Club participation to students in grades other than 3,4, and 5? Was the program targeted at particular grades or something that was integrated more broadly into the school structure? Was this positive or did the approach chosen have limitations or challenges?
4. What elements of the 100 Mile Club do you think were the most successful at your school?
   - - Probe – What made you feel these were successful?
5. What elements of the 100 Mile Club do you think were the least successful at your school?
   - - Probe – What made you feel these were less successful?
6. Can you tell me about any challenges you faced in implementing the 100 Mile Club at your school?
   - - Probe – Were there particular resources or support you could have used or felt you didn’t have?
     - Probe – Was enthusiasm for 100 Mile Club consistent throughout the time you had it? Did it grow or decrease? Was it consistent across both years of the program? If there was a decline, why do you think that might have occurred?
7. How might the program be improved to better engage children?
8. How could the program be improved to better engage teachers, staff, administration or parent volunteers?
9. Looking back on your experience with the 100 Mile Club, what advice would you give to other teachers or schools considering adopting the program in the future?
   - - Probe – Are you planning to use or would you consider using the program again with your students in the future?

***SUMMARIZE THE DISCUSSION.***

Thank you very much for your time today. We have a gift card as a thank you for speaking with us today.

Can I confirm your email address as <email> for the gift card. I am also able to send it to your school to your attention if you prefer.
