# Supplementary material for: The FLEX study school-based physical activity programs – measurement and evaluation of implementation
Source: BMC Public Health. 2019 Jan 16;19:73. doi: 10.1186/s12889-018-6335-3 (PMC6335723; doi:10.1186/s12889-018-6335-3)
Supplement: Supplementary file 2 — FLEX Key Informant Interview Guide – CHALK/Just Move. Key informant interview objective and guide for FLEX Study CHALK/Just Move schools. (DOCX 21 kb) [file 12889_2018_6335_MOESM2_ESM.docx]

**The FLEX Study**

**Key Informant Interviews**

**2015-2017 CHALK/Just Move Teachers**

**Objectives and Guide**

**Objectives:**

- To assess the CHALK/Just Move classroom teacher’s overall satisfaction with the program
- To identify barriers to and enablers of implementation, including which program elements were most successful and to identify ways in which the program could be improved
- To identify other factors in school/classroom environment that may have impacted implementation of the program
- To gain insights into program sustainability and opportunities for scale

**Interview length:** 20-30 minutes

**Introduction (to be read by interviewer):**

Thank you for your involvement with the CHALK/Just Move program in your classroom during the prior (two/one) school year(s). We would like to learn from classroom teachers at schools that implemented Just Move, to understand which aspects of the program were successful and opportunities for improvement. We would also like to identify other factors in the school setting that may have impacted implementing the CHALK/Just Move program. Finally, we would like to get your thoughts on how we might best bring this program to other classrooms.

The interview will take no more than 30 minutes. We will provide a $35 gift card as a thank you for your time. We are asking to record these interviews because we want to make sure we don’t miss any of your valuable feedback. Only Tufts University staff who are involved in this project will have access to the recordings. No identifying information, including your name, will be shared outside our working group, nor will it appear in any publication. You may choose not to answer any of the questions, and you may stop participating at any time. There are no direct benefits to you, but the information you provide will help us to scale and disseminate the CHALK/Just Move program to other schools.

You may contact Dr. Jennifer Sacheck, the Principal Investigator leading this study at (617) 636-3917 for more information. If you have any questions about your rights as a research participant, you should contact Lara Sloboda, the IRB Operations Manager for Social, Behavioral & Educational Research at Tufts University at (617) 627-3276.

**VERBAL CONSENT**

Do you consent to proceed with the interview? Yes_____ No_____

*If yes:* do you agree to be recorded? Yes_____ No_____

*If yes:* we will start the interview now **(turn on recorder).**

*If no:* okay, we will start the interview now **(do not turn on recorder).**

**Discussion topics:**

1. Before I ask about your experience with CHALK/Just Move can you share your thoughts on being asked to use the CHALK/Just Move program in your classroom?
2. We would like to get a sense of your overall satisfaction with the CHALK/Just Move program. Can you say a few words about what it was like having CHALK/Just Move in your classroom during the previous two (one) school years?
3. Now I’d like to dig a bit deeper into some of the specific elements of the program and get a sense of whether you used them in your classroom, and which of them you to be more successful as well as those you may have found to be less successful.
   1. Can you tell me a bit more about how you scheduled the CHALK/Just Move breaks?
      - Probe – Were the breaks regularly scheduled (i.e. same time every day? Following same subject?)?
      - Probe – How long were the breaks typically?
      - Probe – Did you have a daily or weekly goal for the number of breaks?
   2. Can you tell me more about how you used the CHALK/Just Move cards in your classroom?
      - Probe – Did you use mostly blue (high intensity) or mostly yellow cards (yoga poses) or an even mix?
      - Probe – Did you use them in a particular sequence?
      - Probe – If you didn’t use the cards, were there other things you used to guide and/or structure the movements (GoNoodle, etc.)?
   3. Can you tell me more about other elements you may have integrated into the CHALK/Just Move breaks in your classroom?
      - Probe – Did you make the CHALK/Just Move breaks into “working” breaks by integrating them with academic material? If yes, what type of lessons were typically used?
      - Probe – Did you play music during these breaks?
      - Probe – Who led the breaks? Was it typically or always the teacher? Did students lead the breaks? Did you use the opportunity to lead breaks as any kind of reward for your students?
4. Thinking about the various elements of CHALK/Just Move you implemented in your classroom, can you tell me which you found the most successful?
   - - Probe – What made you feel that these were successful?
     - Probe – Was there anything else that we haven’t discussed that you implemented and how did that work for your classroom/students?
5. Thinking about the various elements of CHALK/Just Move you implemented in your classroom, can you tell me which you found the least successful?
   - - Probe – What made you feel that these were less successful?
6. Can you tell me about any challenges you faced in implementing CHALK/Just Move in your classroom?
   - - Probe – Were there particular resources or support you could have used or felt you didn’t have?
     - Probe – Were you and/or your students equally engaged by the CHALK/Just Move program throughout the school year? Did you sense a decline in interest in the program? Why do you think that may have occurred?
     - Probe – [Grade 4 teachers only] Was enthusiasm for CHALK/Just Move consistent across both years of the program? If not, why do you think that may have been the case?
7. How might the program be improved to better engage children?
8. How could the program be improved to better engage teachers?
9. Looking back on your experience with Just Move, what advice would you give to other teachers or schools considering adopting the program in the future?
   - - Probe – Are you planning to use or would you consider using the program again with your students in the future?

***SUMMARIZE THE DISCUSSION.***

Thank you very much for your time today. We have a gift card as a thank you for speaking with us today.

Can I confirm your email address as <email> for the gift card. I am also able to send it to your school to your attention if you prefer.
